# 3D-printed drill guide versus fluoroscopic-guided free-hand technique for pedicle screw insertion in the upper cervical spine: a systematic review and meta-analysis

**DOI:** 10.1007/s12306-024-00879-1

**Published:** 2025-01-12

**Authors:** A. Al-Saadawi, S. Tehranchi, R. Chekuri, A. Oehlen, F. Sedra

**Affiliations:** 1 School of Medicine, Faculty of Medicine and Dentistry, Queen Mary University of London, London, England; 2 Department of Trauma and Orthopaedic Surgery, Barts Health NHS Trust, Royal London Hospital, London, E11BB England

**Keywords:** Upper cervical spine, 3D-printed drill guide, Free-hand technique, Pedicle screw

## Abstract

3D-printed (3DP) drill guides have demonstrated significant potential to accurately guide pedicle screw insertion in spinal surgery. However, their role in the upper cervical spine is not well established. This review aimed to compare the efficacy and safety of 3DP drill guides to the conventional fluoroscopic-guided free-hand technique for pedicle screw insertion in the upper cervical spine. A comprehensive literature search was conducted across five databases (Medline, Scopus, CENTRAL, Web of Science, and Embase). The meta-analysis compared the accuracy of pedicle screw placement, screw placement time, operative time, blood loss, fluoroscopy usage, and post-operative JOA and VAS scores between the two approaches. Seven studies were included in the review, encompassing 386 patients and 1,512 screws. The meta-analysis demonstrated that 3DP drill guides increased the rate of *perfect* screw insertion (OR: 4.34, *P < *0.00001) and lowered the incidence of *moderate* (OR: 0.26, *P < *0.00001) and *poor* (OR: 0.09, *P < *0.00001) screw insertion compared to the free-hand technique. Additionally, operative time (MD: -36.07, *P < *0.00001), blood loss (MD: -83.82, *P < *0.00001), and fluoroscopy usage (MD: -3.47, *P < *0.0001) was significantly reduced in the 3DP cohort. No significant difference was detected in screw placement time (MD: -2.65, *P = *0.07), or post-operative JOA (MD: 0.17, *P = *0.47), and VAS (MD: -0.09, *P = *0.19) scores between the two cohorts. The review demonstrated that 3DP drill guides are a safe and effective tool to assist pedicle screw fixation in the upper cervical spine.

## Introduction

The upper cervical spine, otherwise known as the atlantoaxial spine, consists of the atlas (C1) and axis (C2) vertebrae [1]. Both vertebrae possess unique anatomical features that are crucial for enabling the necessary range of motion to generate neck movements, while also stabilising the head [1, 2]. Additionally, it protects the spinal cord and other critical neurovascular structures, including the vertebral arteries [2]. The atlantoaxial spine is prone to traumatic injuries, often secondary to vehicle accidents, falls, and sports-related trauma [2, 3]. Non-traumatic injuries to this region can stem from tumours, arthritis, and osteoporosis [3]. These injuries can destabilise the atlantoaxial spine, potentially damaging critical surrounding structures, resulting in significant morbidity and mortality [4]. Therefore, safe and efficient management of upper cervical spine injuries is vital for preventing serious complications and preserving neurological function.

Posterior atlantoaxial pedicle screw fixation is a universally accepted technique for managing atlantoaxial instability [5]. Historically, the free-hand technique has been used to guide pedicle screw insertion, relying on the surgeon’s experience and knowledge of relevant anatomical landmarks to determine the optimal screw trajectory point [6]. To enhance the accuracy of free-hand technique, intra-operative fluoroscopy is often used to obtain real-time feedback on screw placement through X-ray imaging [6]. Although considered safe and effective [7], the free-hand technique is significantly more challenging in the upper cervical spine in comparison to other regions. The atlas and axis have narrow pedicles, with studies reporting an average diameter of 4.43 mm [8] and 4.99 mm [9], respectively. Given that the standard screw size used for this procedure is 3.5 mm [10], near-perfect precision is required to avoid complications. Additionally, there is significant anatomical variation in the atlantoaxial spine [11], which further complicates screw placement. Hence, the rate of misplaced screws is high, ranging from 17.5% to 23% [12, 13].

The shortcomings of the free-hand technique have driven efforts to develop a more accurate method that assists surgeons in guiding pedicle screw insertion. Recently, robot-assisted techniques and intra-operative 3D navigation have shown major promise, demonstrating significantly reduced rates of misplaced screws compared to conventional fluoroscopic navigation [14, 15]. However, despite their potential, these approaches have not become standard practice because of several major limitations, including cost and prolonged operation time [16, 17]. Over the years, 3D printing has made significant advancements in the healthcare setting, with its application documented in multiple studies [18, 19]. In spinal surgery, the focus is centred on creating patient-specific 3D models of the spine using pre-operative computed tomography (CT) imaging. These models enable the design of customised drill guides to direct screw placement along the optimal trajectory, thereby ensuring accurate screw placement [20]. Although 3D-printed (3DP) drill guides have demonstrated tremendous success in the field, evidence supporting this approach in the atlantoaxial spine remains limited, as most studies focus on the thoracic, lumbar, and sub-axial cervical spine [21, 22]. Given the unique challenges of pedicle screw placement in the atlantoaxial region, surgeons require the best available support to ensure a safe and efficient operation. Therefore, this systematic review and meta-analysis aimed to evaluate the safety and efficacy of 3DP drill guides compared to the conventional fluoroscopic-guided free-hand technique in guiding pedicle screw insertion in the upper cervical spine.

## Methods

### Protocol and registration

This review was conducted in accordance with the PRISMA (Preferred Reporting Items for Systematic Reviews and Meta-Analyses) guidelines [23]. The protocol was submitted to PROSPERO (International Prospective Register of Systematic Reviews) on March 28th, 2024, and registered on April 3rd, 2024 (PROSPERO ID: CRD42024529931).

### Eligibility criteria

## Inclusion:

- Randomised controlled trials, retrospective cohort studies, or prospective cohort studies.
- Participants are aged 18 years or older with an injury or abnormality in the upper cervical spine (defined as C1 or C2) requiring pedicle screw insertion.
- Studies that included 3DP drill guides as the method of screw insertion in the experimental group, and fluoroscopic-guided free-hand technique as the only employed method in the control group.
- Studies reporting at least one of these outcomes: screw accuracy, screw placement time, operative time, blood loss, fluoroscopy usage, pre-operative and minimum 12-month post-operative JOA scores, or pre-operative and minimum 12-month post-operative VAS scores.

## Exclusion:

- Studies without a comparator control group, case series, case reports, editorials, abstracts, commentaries, expert opinion studies, and review articles.
- Studies with animal or cadaveric participants.
- Studies including techniques other than the free-hand technique in the control group, or additional interventions that may act as confounding factors.
- Studies written in languages other than English.

### Outcomes

The primary outcome of this review was screw accuracy, evaluated using post-operative CT imaging. Secondary outcomes included mean screw placement time, operative time, blood loss, fluoroscopy usage, and both pre-operative and minimum 12-month post-operative Japanese Orthopaedic Association (JOA) and Visual Analog Scale (VAS) scores.

### Information sources and search strategy

Medline, Scopus, CENTRAL, Web of Science, and Embase were searched from their inception until March 29th, 2024, using a search strategy detailed in the supplementary files. The three main keywords used in the search strategy were “cervical spine”, “three-dimensional printing”, and “screw”. The searches were limited to titles and abstracts.

### Study selection

All titles and abstracts were imported into a reference manager and duplicates were screened and removed. The remaining articles were uploaded onto Rayyan [24] and further duplicates undetected by the reference manager were removed. Two reviewers (AAS and SA) screened the remaining articles, tagging each article as “include”, “maybe”, or “exclude”. Articles tagged as “include” by both authors advanced to full-text review, while articles tagged as “include” by only one author or tagged as “maybe” by both authors were resolved through discussions. The remaining articles were excluded from the analysis. A third author (FS) adjourned any disagreements.

### Data extraction

Two independent authors (AAS and SA) extracted all relevant information from the successful articles following the full-text review. The following variables were extracted from each study: (1) author name, (2) date of publication, (3) study design, (4) region of study, (5) cohort size, (6) mean age, (7) sex distribution, (8) pathology, (9) number and location of screws, (10) software and printing technology used for 3D printing, (11) screw accuracy, (12) screw placement time, (13) operative time, (14) blood loss, (15) fluoroscopy usage, (16) pre- and post-operative JOA scores, and (17) pre- and post-operative VAS scores.

Various classification systems have been used across various studies to determine the accuracy of pedicle screw insertion [25–27]. In this review, screw accuracy is categorised into either of three groups: *perfect*, *moderate*, or *poor* accuracy, based on a criterion that incorporates the most commonly used classification systems in modern literature [28]. The criteria used are presented in Table 1.

**Table 1:** Classification criteria for *perfect*, *moderate*, and *poor* screw accuracy

Classification	Description
Perfect	Screw is located entirely within the pedicle
Moderate	Screw violates ≤ 2mm of the bony cortex or less than half of the screw diameter but does not result in complications
Poor	Screw violates ≥ 2mm of the bony cortex, greater than half of the screw diameter, or results in complications directly related to screw location that require revision

### Risk of bias assessment

The risk of bias was assessed using the Newcastle–Ottawa Quality Assessment Scale (NOS) for non-randomised observational studies, which is divided into three components: selection, comparability, and outcome [29]. The criterion for each component can be seen in Table 2. Each criterion is valued at one mark, except for the comparability of cohorts which is valued at two marks, totalling to a maximum score of 9 points. Studies that score 7–9 points are considered “high” quality, 4–6 points are considered “good” quality, and 0–3 are considered “poor” quality [29].

**Table 2:** Components of the Newcastle–Ottawa Quality Assessment Scale

Newcastle–Ottawa Quality Assessment Scale		
Selection	Comparability	Outcome
(1) Representativeness of Experimental Cohort	(1) Comparability of Experimental and Control Cohorts	(1) Assessment of Outcome
(2) Selection of Control Cohort		(2) Adequacy of Follow-up Period for Outcomes to Occur
(3) Ascertainment of Exposure		(3) Duration of Follow-Ups
(4) Study Outcome Not Present at the Start of the Study		

### Statistical analysis

Meta-analyses were performed in accordance with the PRISMA guidelines using the RevMan 5.4 software. A minimum of two studies reporting  the same outcome was required for an individual meta-analysis to be performed. Screw accuracy was reported as dichotomous outcomes and expressed as an odds ratio (OR) with 95% confidence intervals (CI). Continuous data, as in the case of mean screw placement time, operative time, blood loss, fluoroscopy usage, and pre- and post-operative JOA and VAS scores were reported as weighted mean differences (MD) with 95% CI. Heterogeneity was assessed using I^2^ and Chi^2^ statistics where an I^2^ score < 50% or P-value greater than 0.05 respectively was considered insignificant heterogeneity. Pooled results with insignificant heterogeneity employed a fixed-effects model, whereas results with significant heterogeneity utilised a random-effects model.

## Results

**Fig. 1:**
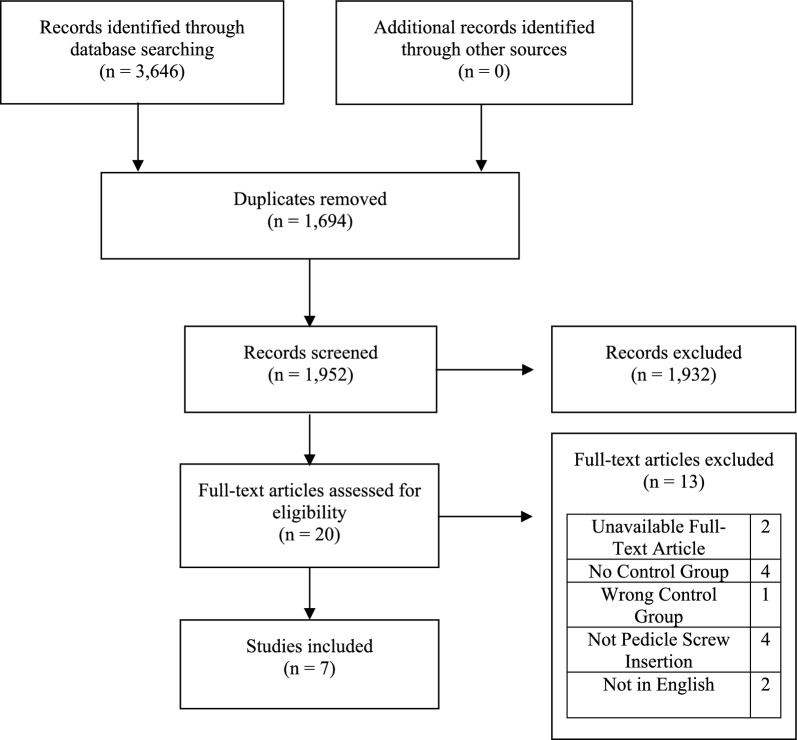
PRISMA flow chart illustrating selection of studies

The search yielded 3,646 results across the five databases and was reduced to 1,952 results after duplicates were removed. After title and abstract screening, 21 articles were deemed eligible and advanced to full-text review. Of these, two studies were excluded because full-text articles were unavailable, and another two were excluded because they were written in Mandarin. Seven studies met the eligibility criteria and were included in the final review (Fig. 1).

### Characteristics of included studies

Seven studies [30–36] were included in the review encompassing 386 patients: 186 patients in the 3DP drill guide cohort and 200 patients in the free-hand cohort. A total of 1,512 pedicle screws were inserted, split into 734 and 778 screws in the 3DP and free-hand cohort, respectively. The mean age ranged from 43.5 – 58 years in the 3DP cohort compared to 42.5 – 58 years in the free-hand cohort. There was a balanced sex ratio in both groups: 49.5% males and 50.5% females in the 3DP cohort, while the free-hand cohort consisted of 51.5% male and 48.5% females. Six studies were based on retrospective cohorts [31–36], and one was a prospective cohort [30]. All seven studies were conducted in China [30–36]. Three studies focused only on upper cervical fractures [31, 32, 35], including two specifically on odontoid fractures [31, 35], while the remaining four studies [30, 33, 34, 36] included patients with a range of pathologies related to the atlantoaxial spine. A detailed summary of the characteristics and outcomes of the included studies are reported in Tables 3 and 4 respectively.

**Table 3:** Summary of characteristics of included studies

Study / Setting	Study Design	Patient Characteristics	Pathology	Intervention Characteristics	3D Technology
Jiang 2017, China [30]	Prospective cohort	Sample Size: 3DP: 25 (16 ♂ and 9 ♀) FHT: 29 (18 ♂ and 11 ♀) Mean Age: 3DP: 43.5 FHT: 46.9	3DP: Congenital dysplasia (11) Traumatic fracture (10) Transverse ligament disruption (2) Rheumatoid disease (2) FHT: Congenital dysplasia (15) Traumatic fracture (7) Transverse ligament disruption (3) Rheumatoid disease (4)	Surgical Technique: Posterior atlantoaxial pedicle screw fixation Screw Location: C1 – C2 Number of Screws: 3DP: 100 FHT: 116	3D Software: Mimics® v17.0 3D Printer: Form1 + Material: Acrylate resin
Li 2021, China [31]	Retrospective cohort	Sample Size: 3DP: 30 (11 ♂ and 19 ♀) FHT: 30 (9 ♂ and 21 ♀) Mean Age: 3DP: N/A FHT: N/A	3DP: Type II Odontoid Fracture (30) FHT: Type II Odontoid Fracture (30)	Surgical Technique: Posterior atlantoaxial pedicle screw fixation Screw Location: C1 – C2 Number of Screws: 3DP: 120 FHT: 120	3D Software: Mimics® v17.0 3D Printer: N/A Material: N/A
Niu 2020, China [32]	Retrospective cohort	Sample Size: 3DP: 12 (9 ♂ and 3♀) FHT: 11 (9 ♂ and 2 ♀) Mean Age: 3DP: 47.8 FHT: 42.5	3DP: Upper cervical fracture (12) FHT: Upper cervical fracture (11)	Surgical Technique: Posterior atlantoaxial pedicle screw fixation Screw Location: C1 – C2 Number of Screws: 3DP: 46 FHT: 42	3D Software: Mimics® v17.0 3D Printer: N/A Material: PLA
Pu 2018, China [33]	Retrospective cohort	Sample Size: 3DP: 25 (11 ♂ and 14 ♀) FHT: 24 (14 ♂ and 10 ♀) Mean Age: 3DP: N/A FHT: N/A	3DP: Upper cervical injuries (25) FHT: Upper cervical injuries (24)	Surgical Technique: Posterior atlantoaxial pedicle screw fixation Screw Location: C1 – C2 Number of Screws: 3DP: 100 FHT: 96	3D Software: Mimics® v17.0 3D Printer: Formlabs 3D Printer Material: N/A
Wang 2019, China [34]	Retrospective cohort	Sample Size: 3DP: 19 (12 ♂ and 7 ♀) FHT: 24 (14 ♂ and 10 ♀) Mean Age: 3DP: 58 FHT: 58	3DP: Atlantoaxial dislocation (5) Atlantoaxial fracture (5) Atlantoaxial fracture and dislocation (9) FHT: Atlantoaxial dislocation (7) Atlantoaxial fracture (8) Atlantoaxial fracture and dislocation (9)	Surgical Technique: Posterior atlantoaxial pedicle screw fixation Screw Location: C1 – C2 Number of Screws: 3DP: 68 FHT: 76	3D Software: Mimics® v17.0 3D Printer: N/A Material: N/A
Xiong 2017, China [35]	Retrospective cohort	Sample Size: 3DP: 13 (7 ♂ and 6 ♀) FHT: 6 (3 ♂ and 3 ♀) Mean Age: 3DP: 46.1 FHT: 48.7	3DP: Old type II odontoid fracture with posterior atlantoaxial dislocation (13) FHT: Old type II odontoid fracture with posterior atlantoaxial dislocation (6)	Surgical Technique: Posterior atlantoaxial pedicle screw fixation Screw Location: C1 – C2 Number of Screws: 3DP: 52 FHT: 24	3D Software: Mimics® v17.0 3D Printer: Photosensitive 3D printer Material: Pangu 4.0
Yang 2018, China [36]	Retrospective cohort	Sample Size: 3DP: 62 (26 ♂ and 36 ♀) FHT: 76 (36 ♂ and 40 ♀) Mean Age: 3DP: 49.8 FHT: 51.3	3DP: Atlantoaxial fracture and dislocation (48) Congenital odontoid non-union (5) Rheumatoid arthritis (6) Other causes of atlantoaxial instability (8) FHT: Atlantoaxial fracture and dislocation (51) Congenital odontoid non-union (7) Rheumatoid arthritis (4) Other causes of atlantoaxial instability (9)	Surgical Technique: Posterior atlantoaxial pedicle screw fixation Screw Location: C1 – C2 Number of Screws: 3DP: 248 FHT: 304	3D Software: Mimics® v17.0 3D Printer: ProJet 360, 3D System Material: N/A

Legend: ♂: Male, ♀: Female, 3DP: 3D-printed drill guide cohort, FHT: Free-hand technique cohort

**Table 4:** Summary of outcomes of included studies

Study / Setting	Screw Accuracy Classification System	Screw Accuracy	Screw Placement Time (min)	Operative Time (min)	Operative Blood Loss (ml)	Number of Fluoroscopy Shots	JOA Score	VAS Score
Jiang 2017, China [30]	Lu et al	3DP: Perfect: 96/100 (96%) Moderate: 4/100 (4%) Poor: 0/100 (0%) FHT: Perfect: 103/116 (88.8%) Moderate: 8/116 (6.9%) Poor: 5/116 (4.3%)	3DP: N/A FHT: N/A	3DP: 171.84 ± 22.46 FHT: 182.76 ± 28.40	3DP: 309.20 ± 33.41 FHT: 322.07 ± 26.51	3DP: 2.76 ± 0.72 FHT: 3.97 ± 0.94	3DP: Pre-Op: 11.16 ± 1.82 Post-Op: 14.16 ± 1.4 FHT: Pre-Op: 11.55 ± 1.88 Post-Op: 14.28 ± 1.62	3DP: Pre-Op: 5.28 ± 0.98 Post-Op: 2.60 ± 0.86 FHT: Pre-Op: 5.34 ± 0.97 Post-Op: 2.93 ± 0.88
Li 2021, China [31]	Gertzbein’s Grading System	3DP: Perfect & Moderate: 118/120 (98.3%) Poor: 2/120 (1.7%) FHT: Perfect & Moderate: 98/120 (81.6%) Poor: 22/120 (18.3%)	3DP: N/A FHT: N/A	3DP: 99.00 ± 24.50 FHT: 124.10 ± 19.30	3DP: 201.00 ± 50.40 FHT: 289.00 ± 41.80	3DP: 2.50 ± 0.80 FHT: 5.90 ± 2.00	3DP: N/A FHT: N/A	3DP: N/A FHT: N/A
Niu 2020, China [32]	Kawaguchi et al	3DP: Perfect & Moderate: 95.7% Poor: 4.3% FHT: Perfect & Moderate: 80.0% Poor: 20%	3DP: N/A FHT: N/A	3DP: 110.00 ± 31.90 FHT: 173.80 ± 53.30	3DP: 159.60 ± 90.10 FHT: 304.00 ± 167.50	3DP: 17.20 ± 8.40 FHT: 30.70 ± 12.70	3DP: N/A FHT: N/A	3DP: N/A FHT: N/A
Pu 2018, China [33]	Kawaguchi et al	3DP: Perfect: 98/100 (98%) Moderate: 2/100 (2%) Poor: 0/100 (0%) FHT: Perfect: 72/96 (75%) Moderate: 16/96 (16.7%) Poor: 8/96 (8.3%)	3DP: 2.10 ± 0.50 FHT: 6.20 ± 0.30	3DP: 106.10 ± 10.90 FHT: 138.20 ± 21.70	3DP: 185.00 ± 59.50 FHT: 311.00 ± 87.40	3DP: 3.10 ± 1.20 FHT: 6.40 ± 2.00	3DP: Pre-Op: 7.64 ± 2.17 Post-Op: 13.71 ± 1.77 FHT: Pre-Op: 7.47 ± 1.84 Post-Op: 13.21 ± 1.58	3DP: Pre-Op: 6.43 ± 0.94 Post-Op: 1.43 ± 0.49 FHT: Pre-Op: 6.32 ± 1.16 Post-Op: 1.79 ± 1.18
Wang 2019, China [34]	Kawaguchi et al	3DP: Perfect: 64/68 (94.1%) Moderate: 2/68 (2.9%) Poor: 2/68 (2.9%) FHT: Perfect: 58/76 (76.3%) Moderate: 12/76 (15.8%) Poor: 6/76 (7.9%)	3DP: 2.20 ± 0.40 FHT: 3.40 ± 0.70	3DP: 197.00 ± 41.00 FHT: 245.00 ± 67.00	3DP: 395.00 ± 64.00 FHT: 552.00 ± 79.00	3DP: 4.60 ± 1.10 FHT: 9.40 ± 2.70	3DP: Pre-Op: 7.60 ± 2.70 Post-Op: 13.90 ± 2.60 FHT: Pre-Op: 7.30 ± 2.60 Post-Op: 13.50 ± 2.20	3DP: Pre-Op: 6.60 ± 1.70 Post-Op: 1.90 ± 0.40 FHT: Pre-Op: 7.30 ± 1.60 Post-Op: 2.20 ± 0.40
Xiong 2017, China [35]	Marcus Ritcher et al	3DP: Perfect: 51/52 (98.1%) Moderate: 1/52 (1.9%) Poor: 0/52 (0%) FHT: Perfect: 21/24 (87.5%) Moderate: 2/24 (8.3%) Poor: 1/24 (4.2%)	3DP: N/A FHT: N/A	3DP: 83.00 ± 20.00 FHT: 116.00 ± 26.00	3DP: 116.00 ± 37.00 FHT: 192.00 ± 69.00	3DP: N/A FHT: N/A	3DP: N/A FHT: N/A	3DP: N/A FHT: N/A
Yang 2018, China [36]	Hojo et al	3DP: Perfect: 219/248 (88.3%) Moderate: 29/248 (11.7%) Poor: 0/248 (0%) FHT: Perfect: 206/304 (67.8%) Moderate: 96/304 (31.6%) Poor: 2/304 (0.7%)	3DP: N/A FHT: N/A	3DP: 105.70 ± 14.60 FHT: 159.40 ± 15.60	3DP: 114.30 ± 14.60 FHT: 164.60 ± 28.40	3DP: N/A FHT: N/A	3DP: Pre-Op: 13.10 ± 2.60 Post-Op: 15.30 ± 2.40 FHT: Pre-Op: 12.70 ± 2.10 Post-Op: 15.10 ± 2.00	3DP: Pre-Op: 3.10 ± 0.40 Post-Op: 1.30 ± 0.40 FHT: Pre-Op: 3.40 ± 0.50 Post-Op: 1.20 ± 0.70

**Legend**: 3DP: 3D-printed drill guide cohort, FHT: Free-hand technique cohort, JOA Score: Japanese Orthopaedic Association Score, VAS Score: Visual Analog Scale Score, Pre-Op: Pre-Operative, Post-Op: Post-Operative

### Risk of bias assessment

The risk of bias based on NOS is summarised in Table 5. Three studies [30, 31, 34] were rated as high quality, while the remaining four studies [32, 33, 35, 36] were considered good quality.

**Table 5:** Summary results of risk of bias assessment using Newcastle–Ottawa Quality Assessment Scale

Authors	Selection				Comparability	Outcome			
	Representative of Exposed Cohort	Selection of Non-Exposed Cohort	Ascertainment of Exposure	Outcome of Interest	Comparability of Cohorts	Assessment	Follow-up Duration	Adequacy of Follow-Up	Total
Jiang 2017	*	*	*	*	*	*	*	*	8
Li 2021	*	*	*	*	*	*	*	-	7
Niu 2020	*	*	*	*	–	*	*	–	6
Pu 2018	*	*	*	*	–	*	*	–	6
Wang 2019	*	*	*	*	–	*	*	*	7
Xiong 2017	*	*	*	*	–	*	*	–	6
Yang 2018	*	*	*	*	–	*	*	–	6

### Accuracy of screw placement

**Fig. 2:**
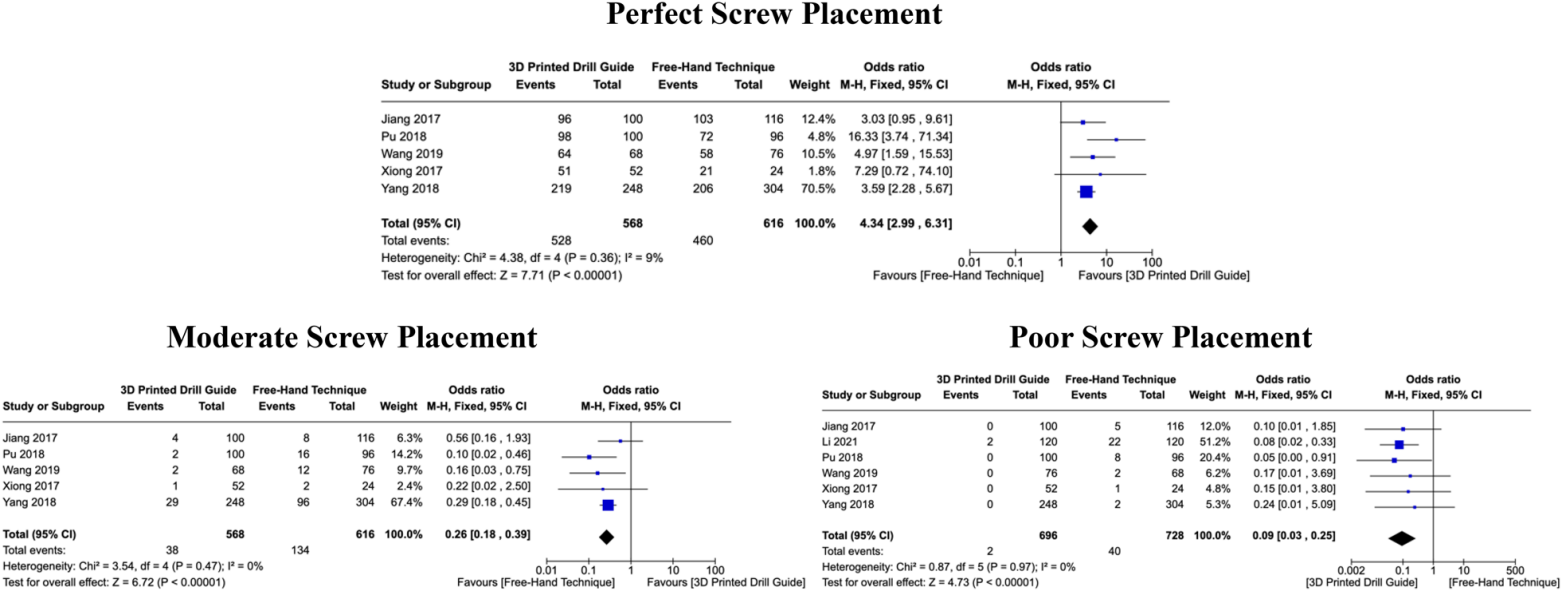
Forest plots comparing *perfect*, *moderate*, and *poor* screw placement between 3DP templates and the fluoroscopic-guided free-hand technique

Five studies reported results sufficient to calculate the OR for *perfect* and *moderate* screw placement, while six studies provided suitable results for *poor* screw placement. Regarding *perfect* screw insertion, there was minimal heterogeneity in results across the five studies (I^2^ = 9%, *P = *0.36), leading to the use of a fixed-effects model. The pooled OR for these studies was 4.34 ([CI: 2.99 – 6.31], *P < *0.00001) in favour of 3DP drill guides. Similarly, no heterogeneity was detected in results for *moderate* (I^2^ = 0%, *P = *0.47) and *poor* screw placement (I^2^ = 0%, *P = *0.97), and a fixed-effects model was also applied for these two datasets. The rate of moderately placed screws was significantly lower in the 3DP drill guide cohort compared to the free-hand cohort (OR: 0.26 [CI: 0.18 – 0.39], *P < *0.00001). Similarly, the incidence of poorly placed screws was significantly lower (OR: 0.09, [CI: 0.03 – 0.25], *P < *0.00001) when using 3DP drill guides compared to the free-hand technique to guide pedicle screw insertion (Fig. 2).

### Screw placement time

**Fig. 3:**
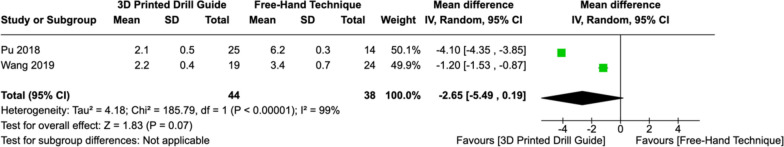
Forest plot comparing screw placement time between 3DP templates and the fluoroscopic-guided free-hand technique

Two studies reported the mean screw placement time in both cohorts. As shown in Fig. 3, there was substantial heterogeneity (I^2^ = 99%, *P < *0.00001) and a random-effects model was deemed most appropriate for analysis. Although screw placement time tended to be lower in the 3DP cohort compared to the free-hand cohort, there was no statistically significant difference in the mean between the two groups (MD: -2.65, [CI: -5.49 – 0.19], *P = *0.07).

### Operative time

**Fig. 4:**
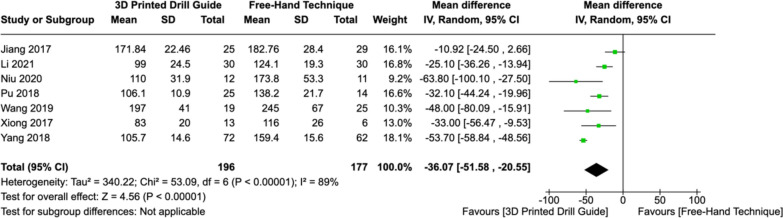
Forest plot comparing operative time between 3DP templates and the fluoroscopic-guided free-hand technique

Seven studies reported the mean operative time in both groups. Among these studies, there was considerable heterogeneity (I^2^ = 89%, *P < *0.00001), necessitating the use of a random-effects model. There operative time was significantly lower (MD: -36.07, [CI: -51.58 – -20.55], *P < *0.00001) using 3DP drill guides compared to the free-hand technique (Fig. 4).

### Operative blood loss

**Fig. 5:**
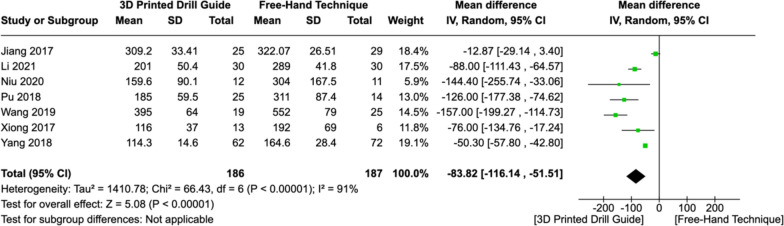
Forest plot comparing operative blood loss between 3DP templates and the fluoroscopic-guided free-hand technique

Seven studies reported the mean operative blood loss in the experimental and control groups. Similar to operative time, a random-effects model was employed due to the substantial heterogeneity amongst studies (I^2^ = 91%, *P < *0.00001). The pooled MD was -83.82 ([CI: -116.14 – -51.51], *P < *0.00001), indicating reduced operative blood loss when using 3DP drill guides compared to the traditional free-hand technique to guide pedicle screw placement in the upper cervical spine (Fig. 5).

### Intra-operative fluoroscopy usage

**Fig. 6:**
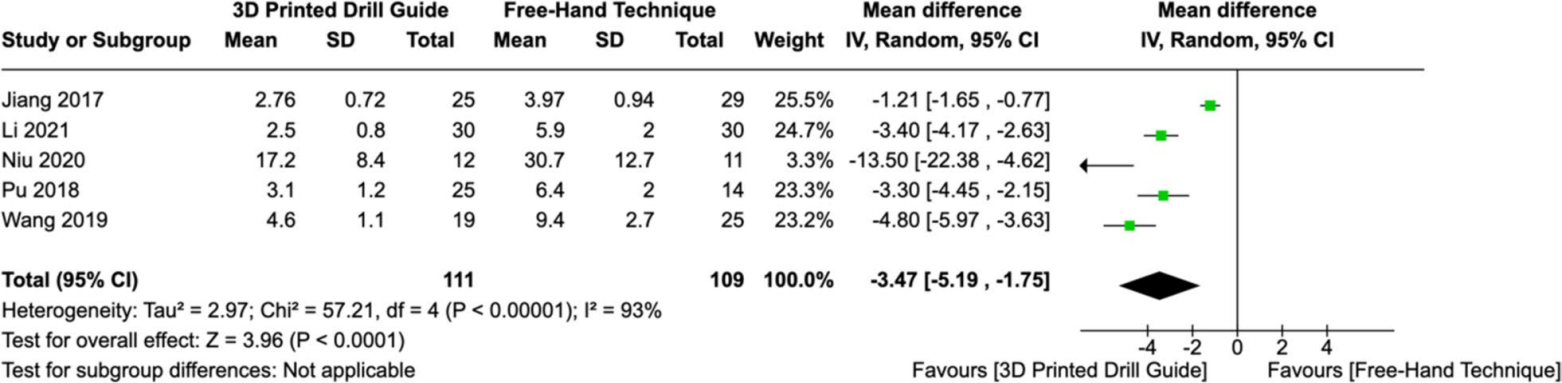
Forest plot comparing intra-operative fluoroscopy usage between 3DP templates and the fluoroscopic-guided free-hand technique

Five studies reported the mean number of fluoroscopy shots used to assist screw insertion in the upper cervical spine. Due to significant heterogeneity (I^2^ = 93%, *P < *0.00001) in the pooled results, a random-effects model was employed. A statistically significant difference was observed in fluoroscopy usage in each cohort (MD: -3.47, [CI: -5.19 – -1.75], *P < *0.0001), with fewer shots required when using 3DP drill guides (Fig. 6).

### Japanese orthopaedic association (JOA) scores

**Fig. 7:**
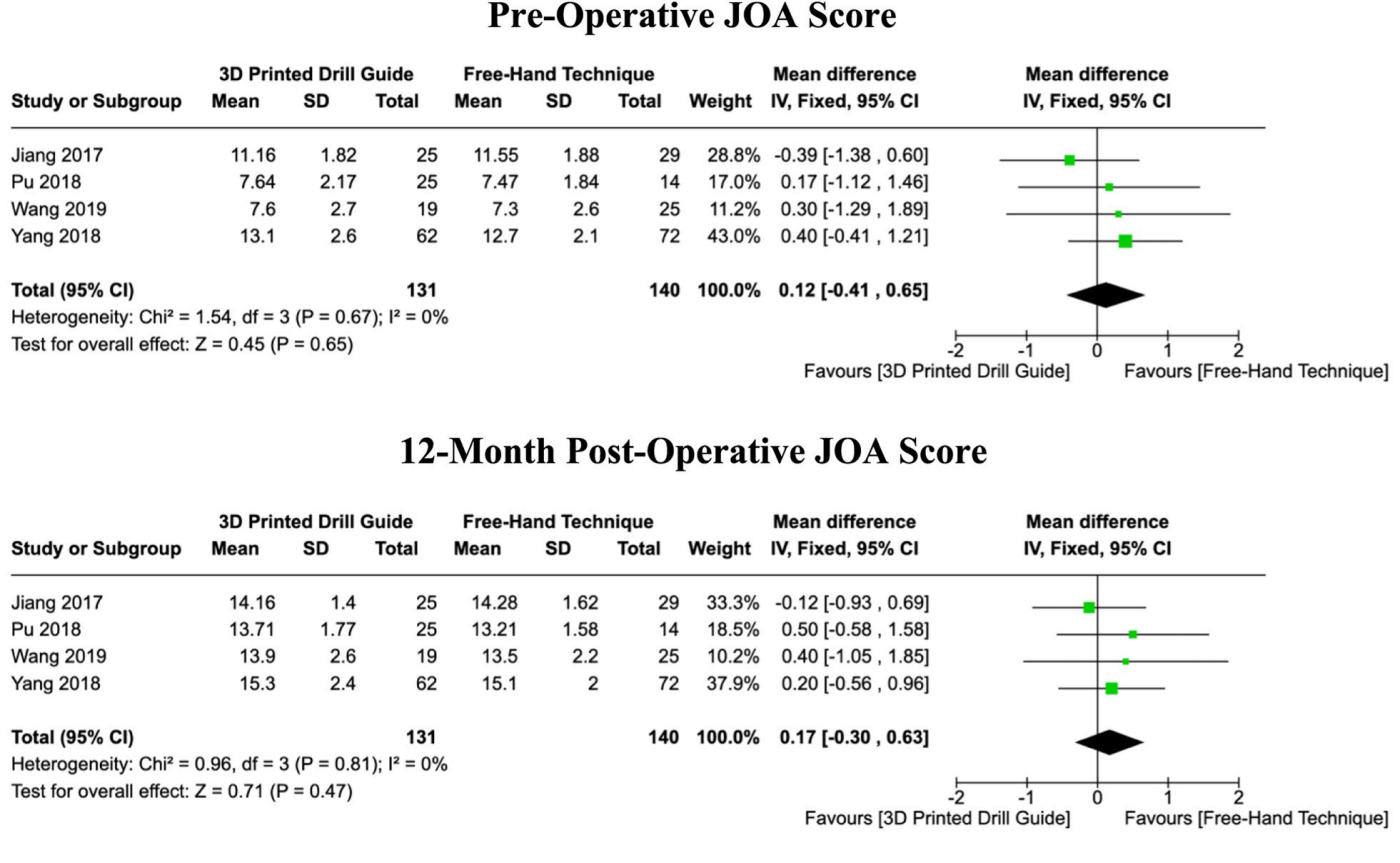
Forest plots comparing pre-operative and 12-month post-operative JOA scores between 3DP templates and the fluoroscopic-guided free-hand technique

Four studies reported both the pre-operative and minimum 12-month post-operative JOA scores. The purpose of pooling the pre-operative JOA scores was to determine whether significant differences existed between the baseline scores of the two cohorts, which may subsequently contribute to potential trends in post-operative scores. A fixed-effects model was used for the pooled analysis of pre-operative scores, as there was no heterogeneity (I^2^ = 0%, *P = *0.67). No significant difference in pre-operative JOA scores was detected between the 3DP and free-hand cohorts (MD: 0.12, [CI: -0.41 – 0.65], *P = *0.65). Furthermore, for post-operative JOA scores, no heterogeneity was observed in results across the four studies (I^2^ = 0%, *P = *0.81), and a fixed-effects model was therefore applied. Similar to the pre-operative scores, no significant difference was detected in the pooled post-operative JOA scores between both cohorts (MD: 0.17, [CI: -0.30 – 0.63], *P = *0.47) (Fig. 7).

### Visual analog scale (VAS) scores

**Fig. 8:**
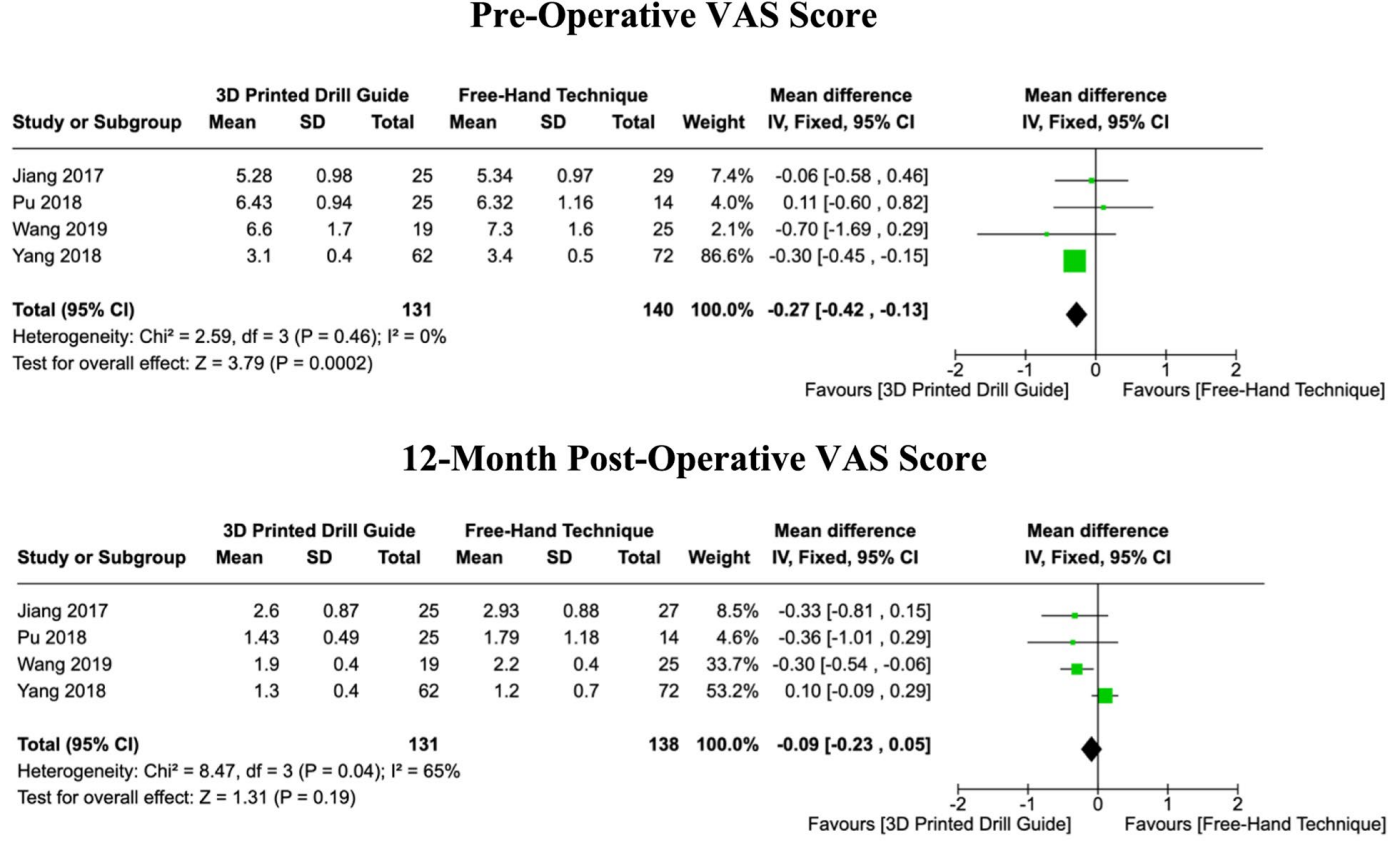
Forest plots comparing pre-operative and 12-month post-operative VAS scores between 3DP templates and the fluoroscopic-guided free-hand technique

Four studies reported both the pre-operative and minimum 12-month post-operative VAS scores. Across these studies, there was no heterogeneity in pre-operative VAS score reporting (I^2^ = 0%, *P = *0.46), prompting the use of a fixed-effects model. There was a significant difference in the pooled pre-operative scores between the two groups, with the 3DP cohort recording lower scores on average (MD: -0.27, [CI: -0.42 – -0.13], *P = *0.0002). In contrast, a random-effects model was utilised for the meta-analysis of post-operative VAS scores due to substantial heterogeneity present among the studies (I^2^ = 65%, *P = *0.04). No significant differences were observed in the post-operative VAS scores between both cohorts (MD: -0.09, [CI: -0.23 – 0.05], *P = *0.19) (Fig. 8).

#### Publication bias

Publication bias was assessed using Egger’s test. There was no significant publication bias in the rate of *perfect* (*P = *0.178), *moderate* (*P = *0.920), or *poor* (*P = *0.244) screw placement. Similarly, no publication bias was detected in the secondary objectives: operative time (*P = *0.287), blood loss (*P = *0.182), intra-operative fluoroscopy usage (*P = *0.087), and post-operative JOA (*P = *0.382) and VAS (*P = *0.378) scores. A formal assessment of publication bias using Egger’s test for the mean screw placement time was not possible due to the limited number of included studies.

## Discussion

This systematic review and meta-analysis compared the accuracy of atlantoaxial pedicle screw placement and various surgical and clinical parameters (mean screw placement time, operative time, blood loss, fluoroscopy usage, JOA, and VAS scores) between patient-specific 3DP drill guides and the conventional fluoroscopic-guided free-hand technique. Overall, the pooled results suggest that 3DP drill guides can significantly improve the accuracy of pedicle screw placement while reducing operative time, blood loss, and fluoroscopy use compared to the free-hand technique.

This meta-analysis demonstrated that the use of 3DP drill guides increased the likelihood of *perfect* screw insertion entirely within the pedicle, and significantly lowered the probability of *moderate* and *poor* screw placement. Supporting our results, *Wallace *et al*.* also reported a higher rate of *excellent* screw placement with 3DP drill guides in a meta-analysis comparing pedicle screw insertion using 3DP drill guides and the free-hand technique across the entire spine [21]. However, only three studies focusing on the atlantoaxial spine were included in the meta-analysis, thereby limiting the validity of the comparisons. Moreover, there was no significant difference in *poor* screw placement between the groups [21]. While the definition of *excellent* screws matched *perfect* screws in this review, *Wallace *et al. defined *poor* screws as a pedicular violation of > 4 mm, differing from our definition of pedicular violation > 2 mm [21]. The discrepancy in classification between the two studies further limits the comparability of findings regarding poorly placed screws. Focusing on the cervical spine, *Azimi *et al*.* conducted a meta-analysis that analysed the application of 3DP drill guides in cervical fusion surgery [37]. In a subgroup analysis consisting of four studies comparing 3DP drill guides to the free-hand technique, a significant improvement in screw accuracy was detected using 3DP drill guides, which is consistent with our findings. However, a similar constraint was present, as three of four studies in the subgroup analysis focused on the atlantoaxial spine, and therefore, the results may not have sufficient power to draw reliable conclusions [37].

Screw placement in the atlantoaxial spine poses unique challenges compared to other regions due to its unique anatomy, significant anatomical variation, and close proximity to critical structures [2, 4, 11] Using a patient-specific 3DP drill guide incorporates the patient’s unique spinal geometrics to flexibly determine the ideal screw trajectory, thereby enhancing the precision of screw placement [20, 38]. Nonetheless, despite the potential advantages that 3DP drill guides may offer, their inherent design can directly lead to screw malposition in certain situations. It relies on strict, predefined channels to ensure that screws are inserted at the optimal angle [39]. While these measures ensure precision, it compromises flexibility, as the screw path cannot be adjusted in cases of a poor fit between the template and spine where unexpected anatomical variances have not been accounted for in the design, resulting in screw malposition [20, 40]. *Deng *et al*.* suggested that 3D templates should not be relied upon in cases of *serious* cervical spinal injuries, such as comminuted fractures, as the drill guides rely on intact anatomical landmarks to accurately guide screw placement [41]. To overcome these design limitations, *Jiang *et al*.* developed a unique template with two location holes and guide rods as opposed to channels to allow for intra-operative adjustment based on the guidance of the surgeon [30]. This highlights the need for additional research to further develop patient-specific 3D templates that will enable greater intra-operative adjustability, while ensuring precision through their individualised nature.

The meta-analysis also highlighted reduced intra-operative blood loss using 3DP drill guides compared to the free-hand technique. By maximising the precision of screw insertion, the risk of compromising surrounding vasculature is reduced [41]. Additionally, patient-specific drill guides reduce the need to manipulate surrounding tissue to achieve an optimal access point, thereby minimising tissue damage and subsequent bleeding [22]. Significant reductions in operative time and fluoroscopy usage associated with 3DP were also noted in this meta-analysis. *Wallace *et al*.* reported similar findings of shortened operative times using 3DP drill guides, solidifying our results [21]. Another meta-analysis by *Liang *et al*.* compared pedicle screw insertion using 3DP drill guides and the free-hand technique in spinal deformity surgery [22]. Interestingly, the study found no significant difference in operative time between both groups [22]. 3DP templates enable shorter operative times through their straightforward application, requiring the surgeon to attach the template to the laminae and follow the predefined channel to guide screw placement [41]. In contrast, the free-hand technique requires a much greater degree of manual navigation and intra-operative assessment from the surgeon to determine the optimal angle for screw insertion, which manifests in longer procedures [6]. Despite the shortened operative time, there is a prolonged pre-operative phase secondary to the rigorous process of producing a patient-specific 3DP template [42]. *Lopez *et al. reported that the production times for 3DP drill guide templates can vary widely, ranging from 9 h to a month [43]. In this context, it may not decrease the overall duration of patient care. Additionally, it may not be feasible in emergency scenarios, which can be seen in the context of spinal cord or other neurovascular compromise. Moreover, there was no significant difference in the mean individual screw placement time between both groups. The confidence interval favoured 3DP drill guides but marginally crossed 0 and therefore was therefore considered non-significant. This outcome could be attributed to the low power of the meta-analysis, stemming from the inclusion of a limited number of studies.

### Strengths and limitations

To the best of our knowledge, this is the first systematic review to compare the effectiveness of patient-specific 3DP drill guides and the fluoroscopic-guided free-hand technique in guiding pedicle screw insertion in the atlantoaxial spine. This research holds crucial clinical significance as it underscores the improved outcomes associated with 3DP drill guides, advocating for a more personalised to spinal surgery.

The central limitation of this review was the limited number of studies included in the final analysis, hindering the overall validity of our results. In addition, there were differences in patient characteristics such as age, sex, and pathology that were not considered in the meta-analysis, which also could have influenced our results to some extent. Moreover, none of the studies were randomised controlled trials, with all seven studies designed as either retrospective or prospective cohort studies. This introduces potential bias and confounding factors that would otherwise not be present in RCTs, thereby further reducing the adequacy of the evidence. This is highlighted by the significant difference detected in pre-operative VAS scores between the 3DP and free-hand cohorts, suggesting a degree of selection bias. Ultimately, this underscores the need for more multi-centre, prospective RCTs with longer follow-up periods to strengthen the evidence-base in this field.

## Conclusion

Overall, 3DP drill guides demonstrated significant improvements in the accuracy of pedicle screw insertion compared to the fluoroscopic-guided free-hand technique. Additionally, they enable a reduction in operative time, blood loss, and fluoroscopy usage. 3DP drill guides are a safe, efficient, and effective tool for assisting pedicle screw insertion in the upper cervical spine. However, evidence in this field is still limited and further multi-centre RCTs are required to strengthen our findings.
